# Protocol for a mixed methods process evaluation of the LinkMM randomised controlled trial “Use of link workers to provide social prescribing and health and social care coordination for people with complex multimorbidity in socially deprived areas”

**DOI:** 10.12688/hrbopenres.13258.1

**Published:** 2021-04-19

**Authors:** Bridget Kiely, Patrick O'Donnell, Vivienne Byers, Emer Galvin, Fiona Boland, Susan M. Smith, Deirdre Connolly, Eamon O'Shea, Barbara Clyne

**Affiliations:** 1 HRB Centre for Primary Care Research, Royal College of Surgeons in Ireland, Dublin 2, Ireland, D02Vn51, Ireland; 2 Graduate Entry Medical School, University of Limerick, Limerick, Ireland, Ireland; 3 Discipline of Occupational Therapy, Trinity College Dublin, Dublin, Ireland, Ireland; 4 School of Business and Economics, National Univeristy of Ireland, Galway, Galway, Ireland

**Keywords:** Multimorbidity, primary care, social prescribing, social deprivation, mixed methods

## Abstract

**Background**

Multimorbidity, defined as two or more chronic conditions is increasing in prevalence and is associated with increased health care use, fragmented care and poorer health outcomes. Link workers are non-health or social care professionals who support people to connect with resources in their community to improve their well-being, a process commonly referred to as social prescribing. The use of link workers in primary care may be an effective intervention in helping those with long-term conditions manage their illness and improve health and well-being, but the evidence base in limited. The LinkMM study is a randomised controlled trial of the effectiveness of link workers based in primary care, providing social prescribing and health and social care coordination for people with multimorbidity. The aim of the LinkMM process evaluation is to investigate the implementation of the link worker intervention, mechanisms of impact and influence of the specific context on these, as per the Medical Research Council framework, using quantitative and qualitative methods.

**Methods**

Quantitative data will be gathered from a number of sources including researcher logbooks, participant baseline questionnaires, client management database, and will be analysed using descriptive statistics. Semi structured interviews with participants will investigate their experiences of the intervention. Interviews with link workers, practices and community stakeholders will explore how the intervention was implemented and barriers and facilitators to this. Thematic analysis of interview transcripts will be conducted.

**Discussion**

The process evaluation of the LinkMM trial will provide important information allowing a more in-depth understanding of how the intervention worked and lessons for future wider scale implementation.

## Introduction

Multimorbidity resulting in fragmented care and poorer health outcomes is a challenge for healthcare systems ^1^. There are limited evidence based interventions aimed specifically at improving outcomes for people with multimorbidity ^2^. Primary care-based link workers providing social prescribing is a potential intervention to improve outcomes for people with multimorbidity, but evidence for their effectiveness is limited ^3^. There is also a lack of clarity on what such interventions involve and a need for process evaluations alongside effectiveness trials ^4^. This paper provides an in-depth description of a link worker intervention and presents the protocol for a process evaluation exploring the implementation, context and mechanisms of action.

### Background

Multimorbidity, defined as two or more chronic conditions, is increasing in prevalence and affects 66.2% of people over 50 years of age attending primary care in Ireland ^5^. Globally, prevalence estimates vary widely due to inconsistent definitions and depending on the specific population being studied. Estimates from two systematic reviews have ranges of between 12.9–95% and 45.1–79% ^6, 7^. Multimorbidity is associated with increased health care usage, fragmented care and poorer health outcomes ^8^. There is also a link between social deprivation and multimorbidity ^9^.

Multimorbidity is recognised as a significant challenge for the health care system as a whole, primary care and areas of social deprivation in particular ^1^. People with multimorbidity who live in deprived areas experience poorer quality of life compared to those with multimorbidity in less deprived areas ^10^. It is not clear why this is, but it could be related to the complex interaction of higher mental health comorbidities and psychosocial stressors. Psychosocial factors such as financial constraints, less social activity and depressive symptoms are related to lower perceived health status in people with multimorbidity ^11^. Mental health comorbidities are twice as common in areas of deprivation compared to less deprived areas ^9^ and anxiety and depression have been identified as barriers to self-management for people with multimorbidity ^12^. Lower self-perceived social support and self-efficacy are also have been shown to negatively impact quality of life for people with multimorbidity ^13– 16^. A recent systematic review of the self-management characteristics of people with complex health needs concluded that tailored self-management support is required for people in areas of socioeconomic deprivation to address the social norms that accept poorer health, social isolation and socioeconomic insecurity ^17^.

A number of complex interventions conducted to support people with multimorbidity have not shown a significant impact on health outcomes or health service utilisation ^2^. One potential intervention to address the complex mix of psychosocial issues and multimorbidity in areas of deprivation is the use of primary care-based link workers providing social prescribing and health and social care coordination. A link worker is a non-health or social care professional who usually has training in motivational interviewing or behaviour change, as well as an extensive knowledge of local community resources. They work with people referred to them by health care services to identify their health and social care needs and support them to access resources within the community to improve their health and well-being ^18^. This process is commonly referred to as social prescribing, which is described as a mechanism for linking people with non-medical sources of support within the community to improve physical, emotional and mental wellbeing. Social prescribing is a general definition, which encompasses activities from providing leaflets on locally available resources, such as libraries and walking groups, to tailored lists of resources, and support to access them from a link worker.

### The LinkMM randomised controlled trial

The LinkMM study is a randomised controlled trial (RCT) of the effectiveness of primary care-based link workers providing social prescribing and health and social care coordination for people with multimorbidity living in areas of deprivation (ISRCTN registration number: ISRCTN10287737). The LinkMM study will take place in 10 urban general practices in Ireland serving areas of deprivation. Participants will be randomised after baseline data collection to an intervention group who will meet with a link worker immediately or join a wait list control who will receive usual care from their general practitioner (GP). Primary outcomes are health related quality of life as measured by EQ-5D-5L ^19^ and mental health as measured by the Hospital Anxiety and Depression Scale ^20^. The economic evaluation will consider costs from the health care system perspective. A full protocol for the RCT and economic evaluation has been published elsewhere ^21^. 

Our intervention is based on the Glasgow Deep End Links Worker model ^22^. The intervention was developed by Deep End GPs, (GPs serving the most deprived areas of Scotland) and draws on the model of community orientated primary care. The rationale behind the link worker was that if people felt supported in their lives, they would be more receptive to information to help them make changes to live well. In principle, social prescribing interventions, such as the link worker intervention, should enable a more holistic response to people’s needs. A realist review of what works in social prescribing identified the link worker as a key component ^4^. Link workers have been piloted in areas of deprivation ^23^, but there remains insufficient evidence to support the effectiveness of link worker interventions on improving health outcomes and reducing health care utilisation ^3^. A number of papers have highlighted that social prescribing interventions often lack a clear description of the assumed underlying pathways and that there is a need to conduct process evaluations alongside effectiveness evaluations to better describe the intervention, the mechanisms of impact and the context in which these have occurred ^4, 24^. This paper will present a protocol for a process evaluation using the Medical Research Council Guidance for Process Evaluations to explore these areas using a mixed methods approach ^25^.

### Description of the intervention

The intervention is a referral of an individual to a primary care-based link worker who provides social prescribing and health and social care coordination. As described previously, the link worker is a non-health or social care worker with skills in motivational interviewing, behaviour change techniques and an in-depth knowledge of the local community resources. 

***Link worker recruitment, training and supervision.*** The link workers will be recruited based on previous experience in the health or social care sector. A person centred, non-judgemental approach and good listening skills have been identified in the qualitative literature as key attributes ^26, 27^. GP practices will be asked to circulate the job adverts amongst their networks and to highlight it to people they believe would be suitable based on their knowledge of community-based services and activities in the local area. Link workers will be recruited, selected, trained and managed however by the research team. The training curriculum is based on the induction for the Glasgow Deep End Links Worker available on the Alliance website ^28^ and will include 40 hours of a blend of face to face interactive training and online learning. During their two-week induction period, the link workers will have a supported introduction to the primary care team and key community stakeholders to start their mapping of community resources. During the intervention, the link workers will have regular supervision from the project manager, who has a background in community research and healthcare management, and monthly learning and support meetings. 

***Link worker activities.*** Once the link worker has received a referral, they will make contact with the participant to arrange an initial meeting. During the initial meeting, the link worker will explore the participant’s health and social care needs and collaborate with them to create individualised goals and identify community resources they can access to improve their health and well-being. The link worker will provide follow up support to achieve the goals and access community resources. It is expected that support will broadly fall into one of four categories; informational (supplying information on resources, directing to websites etc.), instrumental (making an appointment on behalf of a participant or accompanying them to an appointment), appraisal (helping participants to makes changes using behaviour change techniques such as motivational interviewing) or emotional 9listening and encouraging when participants face challenges) ^29^. Face to face or virtual support will be provided in accordance with current public health guidance relating to coronavirus disease 2019 (COVID-19). The nature of community resources recommended by the link worker may also vary depending on availability at the time. Link worker support will be available as much as is deemed necessary between the person and the link worker over a one-month period, at which point the link worker will conduct a final assessment reviewing achievements and sharing outcomes with the person’s GP with their permission. As well as providing support to individuals, the link workers will be tasked with creating a list of available community resources for use by the GP practice in which they are based, updating practice staff about upcoming programmes they may wish to recommend to their patients and building relationships with community resource providers.

Through these actions, it is anticipated that the link workers will reduce psychosocial stresses and improve social connectedness, leading to overall improved quality of life and wellbeing for participants. This in turn should reduce health care utilisation, assuming that these improvements will lead to better capacity to self-manage, improved mental health and reductions in treatment burden. It is also anticipated that by being based in the GP practice, link workers, will improve staff knowledge around community resources and help GPs to respond to psychosocial problems of people with complex health needs, in turn boosting practice staff morale. 

### Logic model

A primary care-based link worker providing social prescribing is a complex intervention, with numerous stages and contextual factors that can influence the implementation and effectiveness ^30^. At the practice level, GPs may differ in how they select people to refer to the link worker and how they accommodate and interact with the link worker. The link workers will have different professional backgrounds and approaches to supporting people with multimorbidity. Community resources will vary between intervention sites. There will therefore be differences in implementation and context between intervention sites. Interactions between intervention participants and each of the stages, (referral, meeting the link worker and engaging with community resources) could determine whether the overall intervention is effective. To summarise the various elements involved, and in keeping with the Medical Research Council (MRC) guidance on process evaluations of complex interventions ^25^, we produced a logic model, based on the Kellogg foundation framework ^31^. This outlines the inputs, activities, outputs, outcomes and impact that are anticipated if the intervention proceeds as planned. Underlying assumptions are also described. The logic model will form the basis for ascertaining if the intervention was implemented as expected and what the potential mechanisms of action were. The logic model for the LinkMM intervention is outlined in Table 1.

**Table 1.** Logic model for the LinkMM intervention.

Assumptions	Inputs	Activities	Outputs	Outcomes	Impact
People with multimorbidity in deprived areas have worse health outcomes because of psychosocial stresses and reduced capacity for self-management GPs can identify people with psychosocial problems that will benefit from a link worker providing social prescribing. People with psychosocial problems will be willing to meet a link worker to discuss them The community has adequate resources to support people referred to them by the link worker Having a link worker in a GP practice will minimise non-attendance rates to the link worker Having a link worker in a GP practice will make practice staff more aware of community resources.	Link worker based in primary care GP referral Community resources	GP select and refer participants Link workers map resources and develop relationships with community resource providers and share this knowledge with practice staff Participants meet with the link worker who through an empathetic approach and time allow an open discussion about psychosocial issues and health concerns Coaching and motivational interviewing approach helps participants set a range of health and social goals The link worker signposts or refers participants to community resources Tailored support from the link worker helps participants achieve their goals/attend community resources including digital access	**For participants:** Attend community resources Engage with online supports and courses Achieve their health and social goals **For practice** **staff :** knowledge of community resources improves	**For participants:** Capability increases Capacity for self- management increases Burden of treatment decreases Activity such as exercise, leisure and self-care increases **For practice staff :** Increased confidence about social prescribing to support people with multimorbidity who present with psychosocial problems	**For participants:** Mental health improves Health related quality of life improves Health care utilisation decreases **For practice staff:** Morale improves Approach to supporting people with multimorbidity becomes more holistic

Finally, the trial is scheduled to take place during the COVID-19 pandemic and there will be varying levels of public health restrictions, which will impact recruitment, meetings with the link worker and the availability of community resources.

### Aims and objectives

The overall aim of the LinkMM process evaluation is to investigate the implementation and mechanisms of potential impact of the link worker intervention, and the influence of the specific context on these, using quantitative and qualitative methods.

The specific objectives are:

1. To describe how the LinkMM intervention was implemented, including selection and recruitment of participants, implementation of the linkworker intervention for individual participants and of the practice and community resource mapping activities of the linkworker.
2. To investigate how participant, practice and local community characteristics affected implementation
3. To examine the impact of the COVID-19 pandemic on recruitment and implementation
4. To consider barriers and facilitators that affected implementation, including recommendations for future implementation
5. To explore presumed mechanisms of impact on participants and practice staff
6. To describe the link worker role and how it evolved during the course of the trial

## Methods

### Overall study design

The process evaluation will use descriptive quantitative and qualitative methods to address the specific objectives outlined and is informed by the MRC guidance for conducting process evaluations ^25^. The process evaluation will run in parallel with the main trial. Detailed methods for each objective are presented in Table 2.

**Table 2.** Detailed methods for each objective.

Objective	Sample	Data sources	Analysis
**1. To describe how the LinkMM** ** intervention was implemented,** ** including selection and recruitment** **of patients, fidelity, dose and reach of** ** the intervention, and the link workers** ** interaction with the practice and** ** community resources**	Recruitment: all participants Implementation: all intervention group participants	Researcher logbooks, participants baseline questionnaire, client management database, survey of link workers, community resource map	Descriptive statistics The relationship between number of meetings with the link worker and primary outcomes will be examined using linear regression as previous studies have suggested this is the case ^29^. Documentary analysis of the community resource maps.
	All link workers (n=10), GPs (n=12), and a purposive sample of community resource providers (n=12)	Semi-structured qualitative interviews	Thematic analysis
**2. To investigate what patient, practice** ** and local community characteristics** ** affected implementation**	Recruitment: all participants Implementation: all intervention group participants Practice and community characteristics: all intervention sites	Researcher logs, patient baseline questionnaire, practice characteristics surveys, client management database and community resource maps.	Descriptive statistics, including exploring variation by intervention site/practice.
**3. To examine the impact of the COVID-19 ** **pandemic on recruitment and** ** implementation**	Recruitment: all participants Implementation: all intervention group participants	Researcher logs and client management database	Descriptive statistics. Recruitment rates, number, and method (face to face or telephone) of meetings will be plotted against the timing of public health restrictions as per the Irish government website.
	All link workers (n=10), GPs (n=12), and a purposive sample of approximately 20 participants and community resource providers (n=12)	Semi-structured interviews	Thematic analysis
**4. To consider barriers and facilitators** **that affected implementation,** ** including recommendations for future** ** implementation**	All link workers (n=10), GPs (n=12), a purposive sample of community resource providers (n=12) and purposive sample of approximately 20 participants	Semi-structured interviews	Thematic analysis
**5. To explore the presumed mechanisms** ** of impact on participants and practices**	At least one GP and one administrative staff member from each participating practice	A community resources knowledge survey emailed to GP practices to assess GP staff knowledge and use of community resources before and after completion of the link worker intervention.	Descriptive statistics
	Purposive sample of approximately 20 participants All GPs (n=12)	Semi-structured qualitative interviews	Thematic analysis
**6. To describe the link worker role and ** **how it evolved during the course of the** ** trial**	All link workers (n=10)	Researcher logs on link worker recruitment	Descriptive statistics
	All link workers (n=10), GPs (n=12), and a purposive sample of community resource providers (n=12)	Semi-structured qualitative interviews	Thematic analysis

### Ethical approval

This study received ethical approval from the Irish College of General Practitioners Research Ethics Committee in August 2019.

### Confidentiality and consent

All participants in the main trial completed informed consent at the time of recruitment to the main trial. They were specifically asked if they consented to doing an interview if selected. Those invited to do an interview verbally confirmed they were still happy to take part at the time of interview. All other study participants (GPs, link workers and community resource providers) will undergo explicit informed consent at the time of invitation to do an interview as part of the process evaluation.

### Study population

Data will be collected from trial participants, link workers, GPs, and community resource providers as appropriate to address the specific objectives, as outlined in Table 2. “Participants” in this protocol refers to participants in the RCT. “Intervention participants” refers to people with multimorbidity who were in the intervention group and had the opportunity to receive ongoing support from the link worker over a one-month period compared to the control group who only met the link worker once. While link workers, GPs and community resource providers are all participants in the process evaluation, they will be named explicitly to avoid confusion.

Quantitative data on recruitment will be gathered on all trial participants and quantitative data on implementation will be gathered on all intervention participants.

During the qualitative data collection, purposive samples of intervention participants and community resource providers will be recruited. Intervention participants will be sampled based on age, gender, geographic spread, timing of intervention (early and later stage to explore changes over time as the link workers become more experienced in their role) and number of meetings with the link worker (including those who did not meet with the link worker or only met with them once). The expected sample is 20 participants, based on a minimum of two interviewees from each intervention site, but may vary depending on heterogeneity of the recruited participants and the point at which data saturation is reached.

A purposive sample of community resource providers suggested by the link worker at each intervention site will be recruited. Link workers will be asked to recommend three providers from each site across a range of services. The link workers will be asked to consider services that they have referred to frequently. These will be purposively sampled to ensure a range of resources, based on the community resource mapping by link workers, is represented. While this sample will be biased in favour of providers with whom the link worker has a good working relationship, there is limited benefit in sampling providers that did not receive any referrals or were inactive during the trial period. The expected sample is ten community resource providers, allowing for one provider from each intervention site.

All ten link workers and all lead GPs will be invited to take part in qualitative interviews.

### Outcome definitions

For the purpose of this evaluation, implementation of the link worker intervention with intervention participants will be considered as a combination of fidelity, dose and reach as defined below.

Fidelity will be defined as the consistency of what is implemented compared with the planned intervention and will be assessed using quantitative data on link worker activity ^25^, including whether the first assessment was completed, if goals were set, and how many and what sort of community resources were recommended. A final assessment will record whether goals were achieved using a standardised goal achievement scale ^32^.

Dose will be defined as the number and nature of meetings with the link worker. It will be assessed via the total number of meetings, method (face to face/telephone etc.) and type of support provided (informational, appraisal, emotional or instrumental) ^29^.

Reach will be assessed by the proportion of intervention participants who meet the link worker at least once and comparing demographic characteristics and reason for selection by the GP of those who met the link worker at least once versus those who were referred to, but did not attend any meeting with the link worker.

The implementation of the practice and community activities will be assessed by the number of practice meetings with the link worker and the depth and breadth of community resources mapped. In the course of their work the link workers will produce an excel spreadsheet of resources available locally that will provide data on the depth and breadth of the community resource mapping. The number of practice meetings will be assessed by a simple count of diary-documented meetings with individual practice team members and meetings with the practice team to discuss resources to support people with multimorbidity and psychosocial needs.

### Data collection

Quantitative and qualitative data will be collected for objectives 1, 3 and 5. Objective 2 will be addressed with quantitative data only, while objectives 4 and 6 will be addressed with qualitative data only.

Quantitative data will be collected from a number of sources, as outlined in Table 2. Data sources for recruitment and intervention implementation include researcher logbooks and reflections, an excel spreadsheet of community resources and the client management database that the link workers will use to support delivery of the intervention. The client management database, using Microsoft Access software, can produce Excel spreadsheets allowing for quantitative analysis of data relevant to fidelity and dose of the link worker intervention with participants.

Practice characteristics will be collected via a paper-based survey at the practice recruitment stage. Data collected will include number of people on the general medical services (GMS) list, the number of people aged over 65, the number of people registered living in disadvantaged areas as defined by the Pobal HP deprivation index. The Pobal HP deprivation index is Ireland’s most widely used social gradient metric and scores each small area (50 – 200 households) in terms of affluence or disadvantage. The index uses information from Ireland’s census, such as employment, age profile and educational attainment, to calculate this score ^33^. The GMS scheme provides medical care to approximately 40% of the Irish population. It is predominantly means-tested and provides eligible patients with free general practitioner visits, free hospital care and free medications (except for a prescription levy, currently €2.50 per item to a maximum of €25). Practice staffing characteristics will include number of whole-time equivalent staff and longevity of service assuming this would affect their knowledge of and relationship with patients.

Trial participant demographics and patient reported outcome measures (PROMs) will be collected at as part of the RCT at baseline, using standardised questionnaires in paper form, self-reported or assisted by a member of the research team if any participants have literacy challenges. Participant characteristics will include age, gender, educational attainment, employment status and socioeconomic status. PROMS include:

- Health related quality of life as measured by EQ-5D-5L ^19^
- Mental health as measured by Hospital Anxiety and Depression Scale ^20^
- Capability and wellbeing as measured by the ICE-CAP A ^34^
- Activities of daily living as measured by the Frenchay Activity Index ^35^
- Self-activation as measured by the Patient Activation Measure ^36^
- Burden of treatment measured by Multimorbidity Burden of Treatment Questionnaire ^37^

Qualitative data will be collected from semi-structured interviews with link workers, lead GPs, community resource providers and intervention participants. Interviews will be conducted by the lead researcher (BK) who is also a GP, audio recorded and transcribed verbatim. Interviews will be conducted over the phone or in a private location convenient to the interviewee, public health restrictions permitting. Telephone interviews can be more convenient and have been shown to be comparable in quality to in-person interviews ^38– 40^. The interview topic guides (included as extended data ^41^) will include questions related to the study objectives.

Topic guides for intervention participants will explore presumed mechanisms of action exploring beliefs about referral to a link worker, support received from the link worker and suitability of, and attendance at, community resources. These areas are informed by a realist review of the literature that sought to understand what works in social prescribing ^4^. For those who didn’t meet with the link worker barriers to attending will be explored such as understanding of the intervention, trust in the referrer, concerns about attending the service in their GP practice etc. and for people who only met once if the quality of the relationship or delivery of the intervention had affected their decision not to continue.

The GP interview topic guide will include questions related to implementation on patient selection and recruitment, how the link worker “embedded” in the practice and the relationships built between link workers, GPs and community resource providers. Questions related to mechanisms of action will explore GPs prior experiences dealing with complex cases and referring to community resources and whether these have changed as a result of the link worker.

The link worker topic guides will include questions exploring prior understanding of the role and how it evolved over the course of the intervention, the underlying assumptions about being embedded in the GP practice, their perception of the participants willingness to engage, if the link worker felt resourced to perform their role and whether suitable community resources were available for onward referral.

Community resource provider topic guides will explore their experiences of the link worker role and the impact of COVID-19 on the services they could provide.

The impact of COVID-19 will be explored in all interviews in terms of the GPs experience of recruiting participants, link workers ability to engage participants and connect them with suitable and accessible resources and community resource providers’ ability to operate, and impact on participants’ experience. Topic guide development has been informed by the four domains of normalisation process theory (NPT); coherence, cognitive participation, collective action and reflexive monitoring ^42^. NPT has been widely used in the evaluation of complex interventions and found to support the identification of barriers and facilitators to implementation at the individual and organisational level ^43^. 

### Public patient involvement

A public patient involvement (PPI) panel comprising of people with multimorbidity supports this study. Members of the panel were asked to comment on the topic guide for participants. Changes were made to the phrasing and ordering of questions, and a question about cost was added. The panel had previously discussed the project as a group with BK and given feedback on information leaflets and questionnaires for the RCT. Further details on the PPI panel is included as extended data. (GRIPP 2 Table)

### Plan of analysis

Appropriate descriptive statistics will be presented for all objectives. For objectives 1 and 2, all data will be examined to see if there is significant variation by intervention site/practice, as outlined in Table 2. All analysis will be conducted in STATA v15 ^44^.

All interviews will be transcribed verbatim and all participant data will be pseudo-anonymised by assignment of a unique study ID. NVivo 10 will be used to assist with organising the data for analysis. A thematic analysis will be conducted following the process described by Braun and Clarke ^28^. Thematic analysis will be conducted by one investigator and crosschecked by members of the research team to increase rigour and the validity of the findings. Semi structured interviews will be thematically analysed through the lens of the logic model to look for concordance and discordance with the assumed mechanisms of impact. The resulting themes will be mapped to the four main constructs of NPT.

The same research team are carrying out the process evaluation and the LinkMM trial. Results of the LinkMM trial, individual or practice level outcomes, will not be known at the time of the interviews with intervention participants, link workers, GPs or community resource providers. Results of the LinkMM trial will be available when analysing implementation data from the client management database. Researchers will not be blinded to the results of LinkMM when analysing the process evaluation data.

### Data synthesis

The quantitative and qualitative data on implementation will be summarised in a narrative synthesis to give an in-depth description of what the link workers did. Areas to be examined in the quantitative analysis of participants and practice characteristics (objective 2) will be informed by the qualitative data on mechanisms of action (objective 5). The results from the main trial will be integrated with data on participants’ and practice characteristics and implementation data to identify factors associated with outcomes of the intervention.

Once all data have been gathered and analysed they will be synthesised under each objective to give an in-depth understanding of whether the intervention was implemented as intended, how it worked or didn’t work, in what context and what lessons can be drawn for system wide implementation if appropriate.

### Data management and availability

Data will be pseudonymised, at the time of collection wherever possible and as soon as possible afterwards in the case of interviews. Data will be stored in a secure server in RCSI. Access to pseudonymised data will be limited to members of the research team and access to identifiable data will be restricted to the data controllers or their nominated deputy. Access to anonymised data will be through a formal request specifying the research purpose to the PI or the ISSDA.

### Dissemination

The results will be published in a peer reviewed journal and will also be presented at a relevant conference and disseminated to policymakers, patients, and the public.

### Study status

Qualitative data collection is near completion and analysis of quantitative data is underway.

## Discussion

Conducting process evaluations is recognised as best practice in the evaluation of complex interventions ^30^. Transparently reporting context helps to improve the internal and external validity of the results of the main trial by demonstrating clearly in what context the intervention was effective or ineffective and whether it might be transferrable to other contexts. Furthermore, if onward spread and adoption is to be achieved, it is important to understand in which contexts it works best ^45^. Our process evaluation will examine practice and community context in detail and specifically the impact of the COVID-19 pandemic and resulting public health restrictions. In the case of a negative trial result it is also important to understand if implementation or the intervention itself failed and if there are any lessons to be learned for modifications to the intervention that could improve implementation ^46^.

Previous evaluations of link worker social prescribing interventions have often had insufficient details on who received what, when and where ^47^. There is limited data on the link worker role and given how central it is to social prescribing interventions, it is important to better understand this ^48^. A review of barriers and facilitators to social prescribing noted that the overall quality of evidence was poor and more research with transparent reporting was required ^49^. Another realist review noted that there was limited data on context, participant health conditions and types of activity to which participants were linked ^50^.

Our process evaluation has a number of strengths that should address some of these outstanding questions about link worker social prescribing interventions and follows best practice in examining implementation, context and mechanisms of action ^25^.

### Strengths

Our process evaluation includes a pre-specified logic model which has informed the objectives of this protocol and which will be used to support thematic analysis of qualitative data. Link worker interventions have generally developed organically with no underlying theory ^24^ and so we have focused on the logic model in the absence of a universal underpinning theory ^25^. We are using the MRC guidance for process evaluations and NPT to increase the rigour of our approach and ensure all relevant elements of implementation are examined that may affect the effectiveness of the intervention and to identify barriers and facilitators to implementation. The client management database developed to support the link workers in delivering the intervention allows for detailed and contemporaneous collection of data on implementation. Qualitative interviews will be conducted with a wide range of stakeholders and findings synthesised to give an in-depth account of implementation, the link worker role, context and the impact of COVID-19 pandemic restrictions.

### Limitations

There is potential that quantitative data on implementation in the client management database may not be completed by the link workers. Assessing the availability of community resources is dependent on the quality of the mapping performed by the link workers. The underlying assumptions of our logic model may be incorrect, making it more difficult to interpret correlations between outputs and intermediary outcomes. The study is not powered to detect differences for all outcomes. The same research team is working on the main trial and the process evaluation, so blinding is not possible.

## Conclusion

Conducting process evaluations of complex interventions is recognised as best practice. This protocol follows best practice guidance and will collect a wide range of quantitative and qualitative data to provide a comprehensive evaluation of the implementation of the intervention. This includes consideration of context, structure, process, fidelity, outcomes and impact. This will allow a better understanding of why the intervention was effective or ineffective and provide recommendations for future roll out of primary care-based link workers providing social prescribing.

## Data availability

The data referenced by this article are under copyright with the following copyright statement: Copyright: ï¿½ 2021 Kiely B et al.

### Underlying data

No data are associated with this article.

### Extended data

Open Science Framework: Protocol for a Mixed Methods Process Evaluation of the LinkMM Randomised Controlled Trial “Use of link workers to provide social prescribing and health and social care coordination for people with complex multimorbidity in socially deprived areas”. https://doi.org/10.17605/OSF.IO/B8NTG ^41^.

This project contains the following extended data:

- GRIPPLInkMMTrial
- LinkMM Interview Topic Guide GP
- LinkMM Interview Topic Guide Patients
- LinkMM Interview Topic Guide Resource Providers

Data are available under the terms of the Attribution-NonCommercial-NoDerivatives 4.0 International license (CC BY-NC-ND 4.0).

## References

[1] Fortin M Soubhi H Hudon C : Multimorbidity's many challenges. *BMJ.* 2007;334(7602):1016–7. 10.1136/bmj.39201.463819.2C 17510108 PMC1871747

[2] Smith SM Wallace E O'Dowd T : Interventions for improving outcomes in patients with multimorbidity in primary care and community settings.Cochrane Library. *Cochrane Database Syst Rev.* 2016;3(3): CD006560. 10.1002/14651858.CD006560.pub3 26976529 PMC6703144

[3] Bickerdike L Booth A Wilson PM : Social prescribing: less rhetoric and more reality. A systematic review of the evidence. *BMJ Open.* 2017;7(4): e013384. 10.1136/bmjopen-2016-013384 28389486 PMC5558801

[4] Lovell R Husk K Blockley K : A realist review and collaborative development of what works in the social prescribing process. *Lancet.* 2017;390:S62. 10.1016/S0140-6736(17)32997-5

[5] Glynn LG Valderas JM Healy P : The prevalence of multimorbidity in primary care and its effect on health care utilization and cost. *Fam Pract.* 2011;28(5):516–23. 10.1093/fampra/cmr013 21436204

[6] Garin N Koyanagi A Chatterji S : Global Multimorbidity Patterns: A Cross-Sectional, Population-Based, Multi-Country Study. *J Gerontol A Biol Sci Med Sci.* 2016;71(2):205–14. 10.1093/gerona/glv128 26419978 PMC5864156

[7] Violan C Foguet-Boreu Q Flores-Mateo G : Prevalence, determinants and patterns of multimorbidity in primary care: a systematic review of observational studies. *PLoS One.* 2014;9(7):e102149. 10.1371/journal.pone.0102149 25048354 PMC4105594

[8] Wallace E Salisbury C Guthrie B : Managing patients with multimorbidity in primary care. *BMJ.* 2015;350: h176. 10.1136/bmj.h176 25646760

[9] Barnett K Mercer SW Norbury M : Epidemiology of multimorbidity and implications for health care, research, and medical education: a cross-sectional study. *Lancet.* 2012;380(9836):37–43. 10.1016/S0140-6736(12)60240-2 22579043

[10] Lawson KD Mercer SW Wyke S : Double trouble: the impact of multimorbidity and deprivation on preference-weighted health related quality of life a cross sectional analysis of the Scottish Health Survey. *Int J Equity Health.* 2013;12:67. 10.1186/1475-9276-12-67 23962150 PMC3765174

[11] Bayliss EA Ellis JL Steiner JF : Barriers to self-management and quality-of-life outcomes in seniors with multimorbidities. *Ann Fam Med.* 2007;5(5):395–402. 10.1370/afm.722 17893380 PMC2000313

[12] Bratzke LC Muehrer RJ Kehl KA : Self-management priority setting and decision-making in adults with multimorbidity: a narrative review of literature. *Int J Nurs Stud.* 2015;52(3):744–55. 10.1016/j.ijnurstu.2014.10.010 25468131 PMC4315694

[13] Fortin M Bravo G Hudon C : Psychological distress and multimorbidity in primary care. *Ann Fam Med.* 2006;4(5):417–22. 10.1370/afm.528 17003141 PMC1578652

[14] Olaya B Domènech-Abella J Moneta MV : All-cause mortality and multimorbidity in older adults: The role of social support and loneliness. *Exp Gerontol.* 2017;99:120–126. 10.1016/j.exger.2017.10.001 28982608

[15] Warner LM Ziegelmann JP Schüz B : Maintaining autonomy despite multimorbidity: self-efficacy and the two faces of social support. *Eur J Ageing.* 2011;8(1):3–12. 10.1007/s10433-011-0176-6 28798638 PMC5547307

[16] Peters M Potter CM Kelly L : Self-efficacy and health-related quality of life: a cross-sectional study of primary care patients with multi-morbidity. *Health Qual Life Outcomes.* 2019;17(1):37. 10.1186/s12955-019-1103-3 30764833 PMC6376655

[17] Gobeil-Lavoie AP Chouinard MC Danish A : Characteristics of self-management among patients with complex health needs: a thematic analysis review. *BMJ Open.* 2019;9(5):e028344. 10.1136/bmjopen-2018-028344 31129599 PMC6538095

[18] Polley MJ Fleming J Anfilogoff T : Making sense of Social Prescribing.University of Westminster;2017. Reference Source

[19] Gusi N Olivares PR Rajendram R : The EQ-5D Health-Related Quality of Life Questionnaire.In: Preedy VR, Watson RR, editors. *Handbook of Disease Burdens and Quality of Life Measures.*New York, NY: Springer New York;2010;87–99. 10.1007/978-0-387-78665-0_5

[20] Brennan C Worrall-Davies A McMillan D : The Hospital Anxiety and Depression Scale: a diagnostic meta-analysis of case-finding ability. *J Psychosom Res.* 2010;69(4):371–8. 10.1016/j.jpsychores.2010.04.006 20846538

[21] Kiely B Clyne B Boland F : Link workers providing social prescribing and health and social care coordination for people with multimorbidity in socially deprived areas (the LinkMM trial): protocol for a pragmatic randomised controlled trial. *BMJ Open.* 2021;11(2):e041809. 10.1136/bmjopen-2020-041809 33526499 PMC7852975

[22] Mercer SW Fitzpatrick B Grant L : Effectiveness of Community-Links Practitioners in Areas of High Socioeconomic Deprivation. *Ann Fam Med.* 2019;17(6):518–25. 10.1370/afm.2429 31712290 PMC6846279

[23] Mercer SW Fitzpatrick B Grant L : The Glasgow ‘Deep End’ Links Worker Study Protocol: A Quasi-Experimental Evaluation of a Social Prescribing Intervention for Patients with Complex Needs in Areas of High Socioeconomic Deprivation. *J Comorb.* 2017;7(1):1–10. 10.15256/joc.2017.7.102 29090184 PMC5556433

[24] Stevenson C Wilson I McNamara N : Social prescribing: A practice in need of a theory. *Br J Gen Pract.* 2019. Reference Source

[25] Moore GF Audrey S Barker M : Process evaluation of complex interventions: Medical Research Council guidance. *BMJ.* 2015;350:h1258. 10.1136/bmj.h1258 25791983 PMC4366184

[26] Moffatt S Steer M Lawson S : Link Worker social prescribing to improve health and well-being for people with long-term conditions: qualitative study of service user perceptions. *BMJ Open.* 2017;7(7):e015203. 10.1136/bmjopen-2016-015203 28713072 PMC5541496

[27] Pescheny J Randhawa G Pappas Y : Patient uptake and adherence to social prescribing: a qualitative study. *BJGP Open.* 2018;2(3):bjgpopen18X101598. 10.3399/bjgpopen18X101598 30564731 PMC6189784

[28] Alliance: Record of Learning; defining the links approach.2017. Reference Source

[29] Langford CP Bowsher J Maloney JP : Social support: a conceptual analysis. *J Adv Nurs.* 1997;25(1):95–100. 10.1046/j.1365-2648.1997.1997025095.x 9004016

[30] Craig P Dieppe P Macintyre S : Developing and evaluating complex interventions: the new Medical Research Council guidance. *BMJ.* 2008;337:a1655. 10.1136/bmj.a1655 18824488 PMC2769032

[31] Kellogg Foundation: Logic Model Development Guide. *Using Logic Models to Bring Together Planning, Evaluation, and Action Michigan*. W. K. Kellogg Foundation;2004;1–71. Reference Source

[32] Turner-Stokes PL : Goal Attainment Scaling in Rehabilitation. A practical guide.Kings College London;2009. Reference Source 10.1177/0269215508101742 19179355

[33] Haase T : The 2016 Pobal HP Deprivation Index (SA). trutzhaase.eu.2016. Reference Source

[34] Al-Janabi H Flynn TN Coast J : Development of a self-report measure of capability wellbeing for adults: the ICECAP-A. *Qual Life Res.* 2012;21(1):167–76. 10.1007/s11136-011-9927-2 21598064 PMC3254872

[35] Schuling J de Haan R Limburg M Groenier KH : The Frenchay Activities Index. Assessment of functional status in stroke patients. *Stroke.* 1993;24(8):1173–7. 10.1161/01.str.24.8.1173 8342192

[36] Hibbard JH Stockard J Mahoney ER : Development of the Patient Activation Measure (PAM): conceptualizing and measuring activation in patients and consumers. *Health Serv Res.* 2004;39(4 Pt 1):1005–26. 10.1111/j.1475-6773.2004.00269.x 15230939 PMC1361049

[37] Duncan P Murphy M Man MS : Development and validation of the Multimorbidity Treatment Burden Questionnaire (MTBQ). *BMJ Open.* 2018;8(4):e019413. 29654011 10.1136/bmjopen-2017-019413 PMC5900423

[38] Fenig S Levav I Kohn R : Telephone vs face-to-face interviewing in a community psychiatric survey. *Am J Public Health.* 1993;83(6):896–8. 10.2105/ajph.83.6.896 8498632 PMC1694719

[39] Novick G : Is there a bias against telephone interviews in qualitative research? *Res Nurs Health.* 2008;31(4):391–8. 10.1002/nur.20259 18203128 PMC3238794

[40] Sturges JE Hanrahan KJ : Comparing Telephone and Face-to-Face Qualitative Interviewing: a Research Note. *Qualitative Research.* 2004;4(1):107–18. 10.1177/1468794104041110

[41] Kiely B : Protocol for a Mixed Methods Process Evaluation of the LinkMM Randomised Controlled Trial “Use of link workers to provide social prescribing and health and social care coordination for people with complex multimorbidity in socially deprived areas”. 2021. 10.17605/OSF.IO/B8NTG PMC10605865 37901156

[42] Murray E Treweek S Pope C : Normalisation process theory: a framework for developing, evaluating and implementing complex interventions. *BMC Med.* 2010;8(1):63. 10.1186/1741-7015-8-63 20961442 PMC2978112

[43] May CR Cummings A Girling M : Using Normalization Process Theory in feasibility studies and process evaluations of complex healthcare interventions: a systematic review. *Implement Sci.* 2018;13(1):80. 10.1186/s13012-018-0758-1 29879986 PMC5992634

[44] StataCorp: Stata Statistical Software:. 15 ed.College Station, TX: StataCorp LLC;2017. Reference Source

[45] Wells M Williams B Treweek S : Intervention description is not enough: evidence from an in-depth multiple case study on the untold role and impact of context in randomised controlled trials of seven complex interventions. *Trials.* 2012;13(1):95. 10.1186/1745-6215-13-95 22742939 PMC3475073

[46] Mann C Shaw ARG Guthrie B : Can implementation failure or intervention failure explain the result of the 3D multimorbidity trial in general practice: mixed-methods process evaluation. *BMJ Open.* 2019;9(11):e031438. 10.1136/bmjopen-2019-031438 31699734 PMC6858134

[47] Bickerdike L Booth A Wilson PM : Social prescribing: less rhetoric and more reality. A systematic review of the evidence. *BMJ Open.* 2017;7(4):e013384. 10.1136/bmjopen-2016-013384 28389486 PMC5558801

[48] Bertotti M Frostick C Hutt P : A realist evaluation of social prescribing: an exploration into the context and mechanisms underpinning a pathway linking primary care with the voluntary sector. *Prim Health Care Res Dev.* 2018;19(3):232–45. 10.1017/S1463423617000706 29215328 PMC5904290

[49] Pescheny JV Pappas Y Randhawa G : Facilitators and barriers of implementing and delivering social prescribing services: a systematic review. *BMC Health Serv Res.* 2018;18(1):86. 10.1186/s12913-018-2893-4 29415720 PMC5803993

[50] Husk K Blockley K Lovell R : What approaches to social prescribing work, for whom, and in what circumstances? A realist review. *Health Soc Care Community.* 2020;28(2):309–24. 10.1111/hsc.12839 31502314 PMC7027770

