# Supplementary material for: Protocol for a mixed methods process evaluation of the LinkMM randomised controlled trial “Use of link workers to provide social prescribing and health and social care coordination for people with complex multimorbidity in socially deprived areas”
Source: HRB Open Res. 2021 Apr 19;4:38. [Version 1] doi: 10.12688/hrbopenres.13258.1 (PMC10605865; doi:10.12688/hrbopenres.13258.1)
Supplement: Peer review history

# Reviewer report: Reviewer response for version 1

**DOI:** 10.21956/hrbopenres.14425.r36585

**Published:** 2023-10-25

**Authors:** Kareem Khan

**Affiliations:** 1 University of Nottingham, Nottingham, UK

This is a clear and concise Protocol for a planned Process Evaluation of the LINKMM RCT. My thoughts for the authors are below:

1. It is logical for the researchers to opt for targeted sampling of the participants of the interviews, but are they not also curious about the overall experience of all participants? I believe that a process evaluation questionnaire for all participants would have been a valuable addition.
2. More information is required on the PPI section (e.g. will they be involved in data analysis?) 
3. It would be useful to include a 'Schedule of LINKMM RCT and process evaluation procedures' figure so we can see at which timepoints the respective data will be collected.

Is the study design appropriate for the research question?

Yes

Is the rationale for, and objectives of, the study clearly described?

Yes

Are sufficient details of the methods provided to allow replication by others?

Yes

Are the datasets clearly presented in a useable and accessible format?

Not applicable

Reviewer Expertise:

Digital intervention, process evaluations, and adolescent mental health.

I confirm that I have read this submission and believe that I have an appropriate level of expertise to confirm that it is of an acceptable scientific standard.

# Reviewer report: Reviewer response for version 1

**DOI:** 10.21956/hrbopenres.14425.r29888

**Published:** 2021-08-16

**Authors:** Alexis Foster

**Affiliations:** 1 School of Health and Related Research, The University of Sheffield, Sheffield, UK

The protocol is very interesting. It is great that there is going to be a trial of social prescribing and accompanying process evaluation. The protocol is well written and clear. There are a few areas where it could be improved. This includes:

1. Further information on the proposed number of interviewees and specified between sample groups.
2. It is not clear what will be included in the researchers' log books or who will be responsible for analysing them and how regularly. 
3. It is not clear how long people will have outcomes collected for - e.g. the intervention is a month, but how long afterwards will post-intervention outcome measures be collected for?
4. Further detail is needed on how the PPI will be involved in the process evaluation, such as, will they be involved in supporting the interpreting of the findings? At present, the only explanation is about involvement when designing the service.
5. It is interesting that the Link Workers were recruited and employed through the study. This may raise questions about sustainability/retention/ incorporating the roles into practice. It would be useful if there was further discussion about how these issues will be explored/considered with the process evaluation. 

Is the study design appropriate for the research question?

Yes

Is the rationale for, and objectives of, the study clearly described?

Yes

Are sufficient details of the methods provided to allow replication by others?

Partly

Are the datasets clearly presented in a useable and accessible format?

Yes

Reviewer Expertise:

Mixed methods evaluation of social prescribing.

I confirm that I have read this submission and believe that I have an appropriate level of expertise to confirm that it is of an acceptable scientific standard.
